# Supplementary material for: Work participation and risk factors for health-related job loss among older workers in the Health and Employment after Fifty (HEAF) study: Evidence from a 2-year follow-up period
Source: PLoS One. 2020 Sep 17;15(9):e0239383. doi: 10.1371/journal.pone.0239383 (PMC7498069; doi:10.1371/journal.pone.0239383)
Supplement: S1 Appendix — (PDF) [file pone.0239383.s001.pdf]

Appendix 1. HEAF questions, response categories, coded analysis variables and reference categories (in italics).

Question	Response categories	Further coding for analysis
At what age do you expect to retire fully?	age in whole years	Proximity to retirement calculated and categorised as follows: 1: less than a year; 2: 1 to <5 years; *3: 5 years to <10 years*; 4: 10 or more years
Do you have any of the following qualifications?	o/gcse levels; a levels; vocational cert; university degree; higher professional qualification	no qualifications/school level vs *vocational training certificate* vs university degree/higher
How much of the household income comes from money you earn in a paid job?	none; less than a quarter; between a quarter and a half; *half or more*	
Is anyone outside your household financially dependent on you?	*no*; yes	
Is your home?	owned outright by you; owned by you with mortgage; rented; rent free	*owned outright* vs mortgage owned vs rented/rent free
How well do you feel you are managing financially these days?	*living comfortably/doing alright;* just about getting by; finding it difficult/very difficult to make ends meet	
Apart from any state pension do you currently receive a private or employer's pension?	no; yes	Combined to: Access only to state pension vs *private pension now or expected in future*
Do you expect to receive a private or employer's pension in the future?	no; yes	
In an average week, outside work, roughly how many hours would you spend doing the following activities?	hours per week physical activities sufficient to make you hot or sweaty	*any hours* vs no hours
	hours per week meeting friends or relatives who do not live in your home	*any hours* vs no hours
Please give your height and your weight	height reported in feet and inches, or centimetres<br>weight reported in stones and pounds, or kilograms	Converted to height in metres and weight in kilograms. Body mass index (BMI) in kg/m^2 was calculated as self-reported weight in kilograms, divided by the square of self-reported height in metres, and categorised as: *normal/underweight (<25kg/m^2)*; overweight (25-29.9kg/m^2); obese/severely obese (≥30kg/m^2).
How much of the following do you drink per week, on average?	beer, cider, lager (pints); wine, sherry (glasses); spirits, liqueurs (measures)	Converted to units of alcohol per week and categorised: *low/no drinker (≤1)*; moderate (2-14); heavy (15+)
Have you ever smoked regularly?<br>If yes, do you still smoke regularly?	no; yes response categories for both questions	Smoker status coded as: *never*; ex; current

Continued on next page

The following items were completed by employed or self-employed people only

Question	Response categories	Further coding for analysis
Is your contract of employment permanent or temporary/renewable?	*permanent*; temporary/renewable; not applicable (self-employed)	
How long have you worked for your present employer?	less than 1 year; 1 to 5 years; *more than 5 years*	
Roughly how many people in total work for your employer?	just you; 2-9; 10-29; 30-499; *500 or more*	
Does your job involve rotating or variable shifts?	often; sometimes; rarely/never	*sometimes/rarely/never* vs often
Does your main job involve night work?	often; sometimes; rarely/never	*sometimes/rarely/never* vs often
In your main job, does an average day at work involve any of the following activities? (all response options No/Yes)	does the job involve kneeling or squatting for longer than 1 hour per day	'Physical work score' ranging from 0 to 6 derived by summing number of affirmative responses. Score set to missing if any component item was missing.
	does the job involve climbing a ladder	
	does the job involve digging or shovelling	
	does the job involve lifting weights of 10kg or more by hand	
	does the job involve standing or walking for more than 3 hours at a time	
	does the job involve hard physical work that makes you hot or sweaty	
How satisfied are you with your job as a whole?	very satisfied; satisfied; dissatisfied; very dissatisfied	*very satisfied/satisfied vs* dissatisfied/very dissatisfied
Provided that you stay well, how secure do you feel our job is?	very secure; secure; rather insecure; very insecure	Each coded to 'secure' (very secure/secure) vs 'insecure' (rather/very insecure) and then combined to contrast 'insecure when well or ill' vs *'secure when well and ill'*
How secure do you feel your job would be if you had an illness that kept you off work?	very secure; secure; rather insecure; very insecure	
If you fell ill and were off work, how long could you get normally paid?	less than one week; 1 to 4 weeks; *1 to 6 months*; more than 6 months; not sure	
If you had a long-term condition would you qualify for an ill-health retirement pension?	no; *yes*; don't know	
Currently, how well do you cope with the physical demand of the job?	easily; with some difficulty; with great difficulty; not coping	*easily* vs at least some difficulty
Currently, how well do you cope with the mental demand of the job?	easily; with some difficulty; with great difficulty; not coping	*easily* vs at least some difficulty
