# Supplementary material for: Work participation and risk factors for health-related job loss among older workers in the Health and Employment after Fifty (HEAF) study: Evidence from a 2-year follow-up period
Source: PLoS One. 2020 Sep 17;15(9):e0239383. doi: 10.1371/journal.pone.0239383 (PMC7498069; doi:10.1371/journal.pone.0239383)
Supplement: S2 Appendix — (PDF) [file pone.0239383.s002.pdf]

Appendix 2. Distribution of job exits between HEAF baseline and 2-year follow-up by sex and reason for leaving employment.

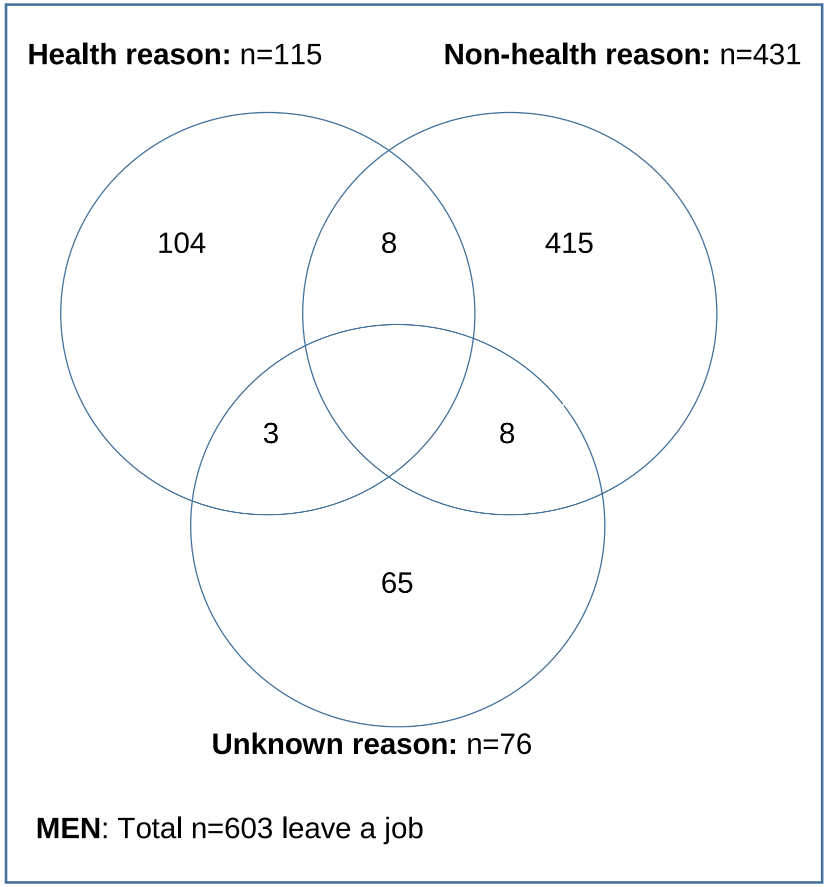

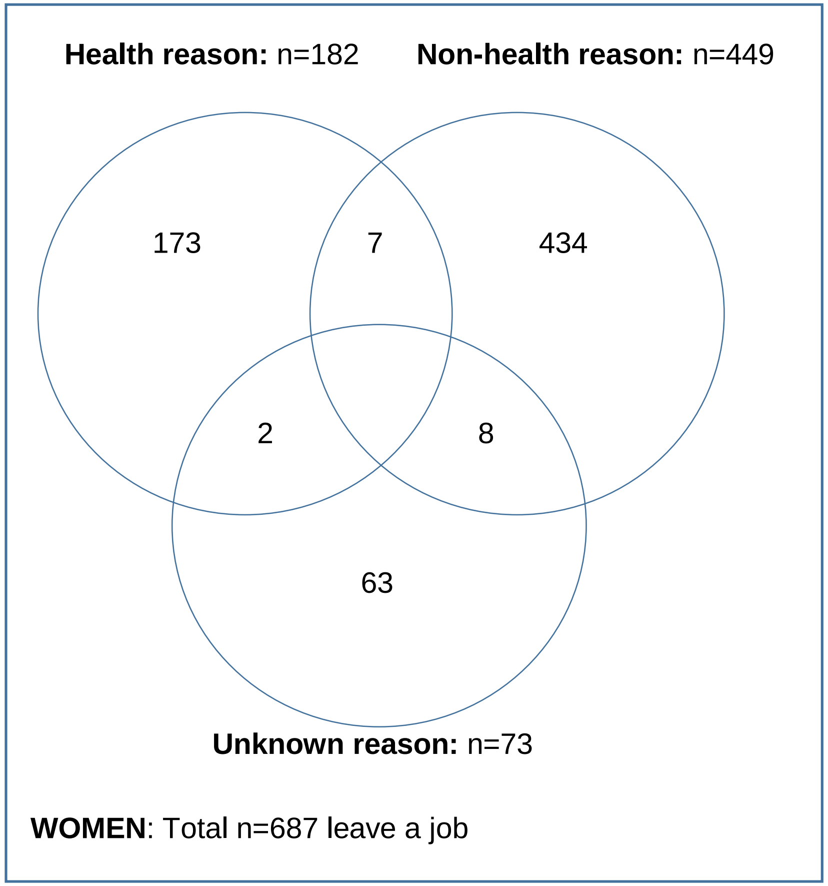

Footnote: 2 men and 8 women reported two health-related job exits; 22 men and 23 women reported two non-health-related job exits; 3 women reported two job exits of unknown reason.
