# Supplementary material for: Work participation and risk factors for health-related job loss among older workers in the Health and Employment after Fifty (HEAF) study: Evidence from a 2-year follow-up period
Source: PLoS One. 2020 Sep 17;15(9):e0239383. doi: 10.1371/journal.pone.0239383 (PMC7498069; doi:10.1371/journal.pone.0239383)
Supplement: S3 Appendix — (3a) and (3b) SOC2010 prevailing* 3-digit job code by sex and work pattern between HEAF baseline and 2-year follow-up. (PDF) [file pone.0239383.s003.pdf]

Appendix 3a. SOC2010 prevailing* 3-digit job code by work pattern between HEAF baseline and 2-year follow-up: men.

N(%) within each SOC2010 3-digit group by work pattern	In work, no exits	HRJL ± other exits	Non-health exit(s)	Unknown exit(s) only
118 Health and Social Services Managers and Directors	2 (0.1)	0 (0.0)	0 (0.0)	2 (3.1)
122 Managers and Proprietors in Hospitality and Leisure Services	7 (0.4)	0 (0.0)	1 (0.2)	2 (3.1)
125 Managers and Proprietors in Other Services	124 (6.6)	3 (2.6)	23 (5.4)	2 (3.1)
Other managers, directors and senior officials	101 (5.4)	4 (3.5)	30 (7.1)	4 (6.2)
212 Engineering Professionals	43 (2.3)	1 (0.9)	18 (4.3)	0 (0.0)
213 Information Technology and Telecommunications Professionals	38 (2.0)	1 (0.9)	19 (4.5)	2 (3.1)
231 Teaching and Educational Professionals	47 (2.5)	3 (2.6)	26 (6.1)	2 (3.1)
242 Business, Research and Administrative Professionals	82 (4.4)	5 (4.4)	20 (4.7)	6 (9.2)
Other professional occupations	99 (5.3)	8 (7.0)	29 (6.9)	2 (3.1)
353 Business, Finance and Related Associate Professionals	29 (1.6)	1 (0.9)	12 (2.8)	2 (3.1)
354 Sales, Marketing and Related Associate Professionals	51 (2.7)	1 (0.9)	20 (4.7)	1 (1.5)
Other associate professional and technical occupations	149 (8.0)	6 (5.2)	32 (7.6)	3 (4.6)
411 Administrative Occupations: Government and Related Organisations	44 (2.4)	3 (2.6)	12 (2.8)	1 (1.5)
Other Administrative and Secretarial Occupations	77 (4.1)	3 (2.6)	18 (4.3)	1 (1.5)
511 Agricultural and Related Trades	50 (2.7)	1 (0.9)	3 (0.7)	2 (3.1)
523 Vehicle Trades	38 (2.0)	6 (5.2)	4 (1.0)	1 (1.5)
524 Electrical and Electronic Trades	47 (2.5)	4 (3.5)	10 (2.4)	2 (3.1)
531 Construction and Building Trades	102 (5.5)	3 (2.6)	7 (1.7)	1 (1.5)
543 Food Preparation and Hospitality Trades	23 (1.2)	4 (3.5)	4 (1.0)	1 (1.5)
Other Skilled Trades Occupations	103 (5.5)	5 (4.4)	16 (3.8)	2 (3.1)
614 Caring personal services	25 (1.3)	2 (1.7)	6 (1.4)	3 (4.6)
Other Caring, Leisure & Other Service Occupations	43 (2.3)	3 (2.6)	3 (0.7)	1 (1.5)
Other Sales & Customer Service Occupations	56 (3.0)	4 (3.5)	14 (3.3)	1 (1.5)
812 Plant and Machine Operatives	59 (3.2)	4 (3.5)	12 (2.8)	0 (0.0)
814 Construction Operatives	23 (1.2)	1 (0.9)	4 (1.0)	3 (4.6)
821 Road Transport Drivers	145 (7.8)	10 (8.7)	20 (4.7)	4 (6.2)
Other Process, Plant and Machine Operatives	58 (3.1)	4 (3.5)	8 (1.9)	0 (0.0)
921 Elementary Administration Occupations	23 (1.2)	4 (3.5)	1 (0.2)	0 (0.0)
923 Elementary Cleaning Occupations	24 (1.3)	4 (3.5)	6 (1.4)	1 (1.5)
926 Elementary Storage Occupations	45 (2.4)	4 (3.5)	5 (1.2)	0 (0.0)
Other Elementary Occupations	67 (3.6)	2 (1.7)	12 (2.8)	1 (1.5)
Unknown	48 (2.6)	11 (9.6)	28 (6.6)	12 (18.5)

Appendix 3b. SOC2010 prevailing* 3-digit job code by work pattern between HEAF baseline and 2-year follow-up: women.

N(%) within each SOC2010 3-digit group by work pattern	In work, no exits	HRJL ± other exits	Non-health exit(s)	Unknown exit(s) only
125 Managers and Proprietors in Other Services	72 (3.6)	3 (1.6)	17 (3.9)	3 (4.8)
Other managers, directors and senior officials	87 (4.4)	5 (2.8)	19 (4.3)	1 (1.6)
223 Nursing and Midwifery Professionals	123 (6.2)	17 (9.3)	20 (4.5)	1 (1.6)
231 Teaching and Educational Professionals	122 (6.2)	19 (10.4)	48 (10.9)	7 (11.1)
242 Business, Research and Administrative Professionals	34 (1.7)	3 (1.7)	13 (2.9)	1 (1.6)
Other professional occupations	121 (6.1)	14 (7.7)	32 (7.2)	0 (0.0)
323 Welfare and Housing Associate Professionals	55 (2.8)	5 (2.8)	16 (3.6)	0 (0.0)
354 Sales, Marketing and Related Associate Professionals	28 (1.4)	8 (4.4)	6 (1.4)	0 (0.0)
Other associate professional and technical occupations	119 (6.0)	6 (3.3)	28 (6.3)	1 (1.6)
411 Administrative Occupations: Government and Related Organisations	58 (2.9)	9 (5.0)	9 (2.4)	0 (0.0)
412 Administrative Occupations: Finance	93 (4.7)	5 (2.8)	15 (3.4)	2 (3.2)
415 Other Administrative Occupations	140 (7.1)	10 (5.5)	38 (8.6)	4 (6.4)
421 Secretarial and related occupations	135 (6.8)	9 (5.0)	33 (7.5)	3 (4.8)
Other Administrative and Secretarial Occupations	64 (3.2)	3 (1.7)	15 (3.4)	2 (3.2)
Skilled Trades Occupations	67 (3.4)	4 (2.2)	8 (1.8)	3 (4.8)
612 Childcare and Related Personal Services	117 (5.9)	8 (4.4)	14 (3.2)	1 (1.6)
614 Caring Personal Services	113 (5.7)	13 (7.1)	23 (5.2)	7 (11.1)
Other Caring, Leisure & Other Service Occupations	61 (3.1)	6 (3.3)	7 (1.6)	3 (4.8)
711 Sales Assistants and Retail Cashiers	121 (6.1)	8 (4.4)	29 (6.6)	4 (6.4)
Other Sales & Customer Service Occupations	44 (2.2)	4 (2.2)	12 (2.7)	3 (4.8)
Process, Plant and Machine Operatives	28 (1.4)	3 (1.7)	2 (0.5)	1 (1.6)
923 Elementary cleaning occupations	54 (2.7)	7 (3.9)	12 (2.7)	1 (1.6)
Other Elementary Occupations	87 (4.4)	5 (2.8)	10 (2.3)	1 (1.6)
Missing	38 (1.9)	8 (4.4)	16 (3.6)	14 (22.2)

Footnote to appendices 3a and 3b:
*Job codes are those prevailing at the time of the first job exit of the type indicated.
Job code for first reported job between baseline and 2-year follow-up is tabulated for people in work with no exits.
HRJL: health-related job loss
