# Supplementary material for: Work participation and risk factors for health-related job loss among older workers in the Health and Employment after Fifty (HEAF) study: Evidence from a 2-year follow-up period
Source: PLoS One. 2020 Sep 17;15(9):e0239383. doi: 10.1371/journal.pone.0239383 (PMC7498069; doi:10.1371/journal.pone.0239383)
Supplement: S1 File — (DOCX) [file pone.0239383.s004.docx]

**FORM B**


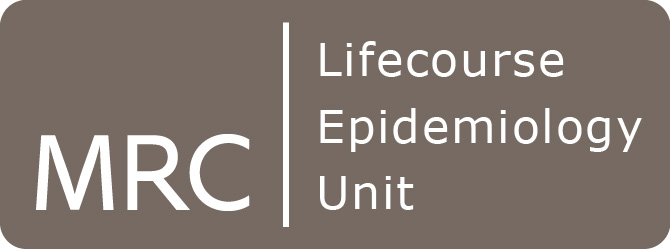


SERIAL NO:

**CONSENT FORM (Version 2: 07/08/2012)**

**Health & Employment After Fifty (the HEAF Study)**

|  | *Please tick boxes* |
| --- | --- |
| I am happy for the MRC research team to send me further (shorter) questionnaires annually by post over the next three years. |  |
| My address for this purpose is:  ........................................................................................................  ........................................................................................................  ........................................................................................................  Postcode ................................................  Mobile phone no. .............................................. *(only to be used if we lose touch)* |  |
| I agree that the MRC research team may have ongoing access to anonymised information from my NHS health records covering:   - consultations or treatments in hospital or in the GP surgery for the following health problems: rheumatic disorders, diabetes, epilepsy, asthma, chronic bronchitis/emphysema, cancer, heart problems, mental illness and injury at work - health problems requiring admission to hospital - height, weight, smoking and alcohol |  |
| I understand that I am free to withdraw this consent at any time, by writing to the study team at Southampton General Hospital. |  |

*Data protection*

*I understand that all information collected about me during my participation in this study will be stored in locked cabinets in a secure building or on a password protected computer, and that this information will be available only to the small study team, and used only for the purpose of this study. All files containing identifying information (names and addresses) will be kept separate from those containing other information about me, which will be identified only by a coded serial number. No information from the study will be published in a form that could lead to the identification of individuals.*

Title ............. Name ........................................................................................

Signature ........................................................................................................

Date .......................................................
