# Supplementary material for: Work participation and risk factors for health-related job loss among older workers in the Health and Employment after Fifty (HEAF) study: Evidence from a 2-year follow-up period
Source: PLoS One. 2020 Sep 17;15(9):e0239383. doi: 10.1371/journal.pone.0239383 (PMC7498069; doi:10.1371/journal.pone.0239383)
Supplement: Attachment — Submitted filename: Response to reviewers.docx [file pone.0239383.s005.docx]

**PONE-D-20-01352
Work participation and risk factors for health-related job loss among older workers in the Health and Employment after Fifty (HEAF) study: evidence from a two year follow-up period. PLOS ONE**

*Editor’s comments:
Thank you for submitting your manuscript to PLOS ONE. After careful consideration, we feel that it has merit but does not fully meet PLOS ONE’s publication criteria as it currently stands. Therefore, we invite you to submit a revised version of the manuscript that addresses the points raised during the review process. The manuscript touches upon interesting and timely aspects, but suffers from a number of weaknesses that have to be thoroughly and fully overcome before any further consideration can be given to it.*

We thank the Editor for their feedback. We are very grateful for the opportunity to revise our manuscript for further consideration for publication in PLOS ONE. We have been careful to respond to all of the reviewers’ comments and feel that our manuscript is improved as a result. We thank the reviewers for their insights and constructive critique of our work. We sincerely hope that our revised manuscript (tracked and unmarked versions) and our point by point response to the reviewers’ comments are satisfactory and mean that our manuscript is now suitable for publication in an esteemed journal such as PLOS ONE.

*Journal Requirements:*

*When submitting your revision, we need you to address these additional requirements.*

*1. Please ensure that your manuscript meets PLOS ONE's style requirements, including those for file naming.*

Thank you for directing us to these style guidelines. We trust that we have been attentive to all of the formatting requirements for the title page, author affiliations, formatting of the main body of the paper and file naming. As directed we have prepared marked-up and unmarked versions of our manuscript.

*Technical points:
While revising your submission, please upload your figure files to the Preflight Analysis and Conversion Engine (PACE) digital diagnostic tool,* [*https://pacev2.apexcovantage.com/*](https://eur03.safelinks.protection.outlook.com/?url=https%3A%2F%2Fpacev2.apexcovantage.com%2F&data=01%7C01%7Ches%40mrc.soton.ac.uk%7Cb09526599db741d61d4708d808515186%7C4a5378f929f44d3ebe89669d03ada9d8%7C0&sdata=hlfNKQJMjYFvl3QNUbNTq1%2F8MhZQ57v28T4VZb1abi0%3D&reserved=0)*. PACE helps ensure that figures meet PLOS requirements.*

We confirm that we have used PACE to ensure that Fig1.tif and Fig2.tif meet PLOS requirements.

*Please provide additional details regarding participant consent. In the ethics statement in the Methods and online submission information, please ensure that you have specified (a) whether consent was informed and (b) what type you obtained (for instance, written or verbal, and if verbal, how it was documented and witnessed).*

Apologies for omitting this information previously. We have included the following statement at the end of the ‘Population’ section at the start of the Methods section of the paper: “When they returned their baseline questionnaire, all participants gave written informed consent to participate in the study and to be sent annual follow-up questionnaires.”

*We note that you have indicated that data from this study are available upon request. PLOS only allows data to be available upon request if there are legal or ethical restrictions on sharing data publicly. In your revised cover letter, please address the following prompts: a) If there are ethical or legal restrictions on sharing a de-identified data set, please explain them in detail (e.g., data contain potentially identifying or sensitive patient information) and who has imposed them (e.g., an ethics committee). Please also provide contact information for a data access committee, ethics committee, or other institutional body to which data requests may be sent.*

The covering letter for our resubmission includes the following sections and we have also expanded the Data Access section in our manuscript:

“We were asked to use this covering letter to explain why we cannot freely share a de-identified data set. This restriction arises from the wording of the data protection statement in the original consent form that the participants signed, dated and returned with their baseline questionnaire. The wording was as follows: *I understand that all information collected about me during my participation in this study will be stored in locked cabinets in a secure building or on a password protected computer, and that this information will be available only to the small study team, and used only for the purpose of this study.* (We have uploaded a copy of the full consent form with our resubmission via the PLOS ONE online system.)

We fully acknowledge that publicly funded research should be accessible through appropriate channels to bone fide researchers. As such, the study metadata are already posted on the UK data service Reshare as a requirement by ESRC who funded part of the HEAF study: [http://reshare.ukdataservice.ac.uk/852663/](https://eur03.safelinks.protection.outlook.com/?url=http%3A%2F%2Freshare.ukdataservice.ac.uk%2F852663%2F&data=01%7C01%7Ches%40mrc.soton.ac.uk%7C9fb883b8df624c6b930b08d811ed3b55%7C4a5378f929f44d3ebe89669d03ada9d8%7C0&sdata=v8hJhLp0pxqdSHzl1oNKSy3jsE4JrdXHX%2BRA3MhgYHs%3D&reserved=0). We are also in the final stages of preparing the study metadata to comply with the requirements of the CLOSER Discovery Platform which will markedly improve the discoverability of the data: <https://discovery.closer.ac.uk/>. CLOSER brings together world-leading longitudinal studies with participants born throughout the 20th and 21st centuries; we are privileged that HEAF has been invited to join CLOSER and delighted that it is being recognised as a key UK longitudinal study in this way.

In addition to these online metadata resources, the Data Access section of our manuscript states that researchers may also access HEAF data by applying to work collaboratively with the study team. Enquiries should be directed to the HEAF principal investigator in the first instance (Prof Karen Walker-Bone, [kwb@mrc.soton.ac.uk](mailto:kwb@mrc.soton.ac.uk), Director of the MRC Versus Arthritis Centre for Musculoskeletal Health and Work, MRC Lifecourse Epidemiology Unit, University of Southampton).”

*Additional Editor Comments (if provided):
The reviewers identified several critical shortcomings in the manuscript, besides some interesting strengths. That said, this invitation to revise the manuscript is a single opportunity to fix all the problems identified in both reviews.*

As noted above, we thank the Editor for their feedback. We are very grateful for the opportunity to revise our manuscript for further consideration for publication in PLOS ONE. We have done a substantial amount of work and been careful to respond to all of the reviewers’ comments and feel that our manuscript is improved as a result. We thank the reviewers for their insights and constructive critique of our work. We sincerely hope that our revised manuscript (tracked and unmarked versions) and our point by point response to the comments are satisfactory and mean that our manuscript is now suitable for publication in an esteemed journal such as PLOS ONE.

*Review Comments to the Author*

***Reviewer #1:*** *The manuscript, “Work participation and risk factors for health-related job loss among older workers in the Health and Employment after Fifty (HEAF) study: evidence from a two year follow-up period” investigates risk factors for health-related job loss (HRJL) among a cohort study of older adults in England, aged 50-64 years at baseline. In the opinion of this reviewer, the manuscript has a potential to make a contribution to the field of public aging and health, but concerns (see below) must first be addressed. This review focuses on conceptualization of approach and communication of key findings.*

We are delighted that the reviewer recognises the value of our work and grateful for their detailed critique of our manuscript. As detailed below, we have made every effort to address the reviewer’s comments and feel that our manuscript is much improved as a result. *Abstract:
1. Because older age thresholds generally begin at age 65 (and the included age group of the study is 50-64), this reviewer recommends changing “older adults” to “middle aged and older adults” or simply “adults aged 50-64 (page 2, line 46 and throughout manuscript).*

Thank you for this comment. We agree that people who are 50-64 years of age are not ‘older adults’ but we have chosen to use the term “older workers” for people aged > 50 years in line with the current World Health Organisation official definition. Therefore, we would like to retain reference to them as ‘older workers’ where appropriate throughout the manuscript. In accordance with the reviewer’s comment however, we have edited line 50 of the Abstract to read: ‘HEAF is a population based cohort study of adults in England (aged 50-64 years at baseline) who provided information…’ We have also edited the start of our Methods section to read: ‘As described previously, the HEAF study follows a large population-based cohort of adults in England (aged 50-64 years at baseline)’.

*Introduction:
2. Avoid beginning paragraphs with “however” and “moreover” (page 4, lines 74, 84).*

Thank you for this comment, we have edited the text as suggested and also combined the second and third paragraphs of the Introduction.

*3. At what point might physical or mental strain have deleterious consequences on health? Are individual protected from age 20-40 and unprotected in later life? This reviewer recommends bringing in outside citations and removing the terms “is unlikely to be exactly the same in later life”, which seems assumptive and somewhat vague. It’s also important to note the many potential positive impacts of work on physical, mental, cognitive, and social health and well-being. Many individuals thrive in work later into their 60s, 70s, and 80s and that should also be recognized here. Also, beyond the “dependency ratio”, older worker have a great deal to contribute. The authors might consider acknowledging how older workers increase the diversity of the workforce and also increasingly reflect the consumer base.*

Thank you for these comments and apologies that our choice of wording gave the impression that individuals would be protected from age 20-40 and unprotected in later life. There is, of course, nothing special about the 20-40 year age range and there are occupational exposures that can exert deleterious impacts on health or wellbeing throughout the lifecourse with either immediate or accumulated consequences later in life (e.g. heavy manual labour involving kneeling, lifting and squatting causing joint pain but eventually leading to established osteoarthritis or joint failure, or exposure to occupational chemicals or dust causing short term respiratory aggravation but longer term increased risk of lung and nasal cancers). We simply meant to raise the point that what is acceptable at work without deleterious consequence to health or wellbeing will not be homogenous throughout the lifecourse.

In light of this, we have edited our Introduction quite extensively and brought in references by Nazroo and Round. We hope we now make it clear that capability to remain in work to older ages accumulates across the lifecourse and varies individually, regionally, and between communities. As such, macro level measures to extend working lives very clearly run the risk of widening the disability employment gap and social inequalities. The second paragraph of our Introduction now reads (please note that references are included in the main tracked changes and clean versions of our manuscript):

‘Macro level measures to extend working life may however be limited in their efficacy and run the risk of widening social inequalities and the disability employment gap because they do not recognise the complex individual level barriers and facilitators that accumulate throughout the lifecourse to affect a person’s ability to extend their working life. In particular, the capability of women to work to older ages is an especially pertinent issue in the light of the recent harmonisation of the pension age for men and women in many European countries. Government policies do not reflect that work is different for men and women across the lifecourse and that, in general, women’s work is more likely to be insecure, part-time, poorly remunerated, and more likely to be dovetailed with family and caring responsibilities. Moreover, although many people thrive on work and will continue to reap its physical, mental, social and financial benefits at older ages, it is not universally feasible to maintain all types of occupations well into the seventh or eighth decades of life and this capability varies not only individually but also regionally and between communities. Different occupations are associated with variable levels of physical and/or mental strain on employees and what is acceptable without deleterious consequence to health or wellbeing will change throughout the working lifecourse. Low-paid older workers who are employed in manual occupations, or who experience disability or chronic morbidity, may face the double jeopardy of needing to earn for longer but being unable to remain in their established job.’

*Methods:
4. What is the rationale for choosing the examined criteria over others? It would be helpful if these factors were explained in a more structured or cohesive way, ideally with a theoretical model, with relevant literature on prior findings regarding the relationship between these factors and HRJL. There are many covariates and the rationale for inclusion should be more clearly stated – a more focused analysis would greatly improve the readability of this paper and its tables (Descriptive tables should fit on one page). Also, was it possible to examine chronic illness co-morbidity or disability/functional limitations?*

We thank the reviewer for these comments which tie in with those from reviewer 2 pertaining to our Introduction. As also noted in our response to reviewer 2 below, our principal focus is on health-related job loss and our literature review focussed on this outcome. We have now amended our Introduction to make this clear, we have referenced previous work in this general topic area but have clarified that there are no previous studies which have explored a comprehensive set of risk factors for health-related job loss in a UK setting. Every country is unique in terms of its political and social contexts, as such, although some findings may be generalizable between different countries, we feel that our UK based study is extremely important and has the ability to greatly inform the ongoing debate on policies designed to extend working lives.

We appreciate that our analysis considered a comprehensive range of socio-demographic, lifestyle and employment characteristics. As outlined in our revised Introduction, although many studies have investigated the links between assorted subsets of these characteristics as risk factors for health-related job loss, few have taken a comprehensive approach to their analysis and certainly no prior study based in the UK. We recognise that some of our tables are quite long but we were mindful that we wanted to present all of our results to the reader and did not want to cherry-pick and focus our analyses on just the factors that remained important in our final models; we felt that this would have been disingenuous and would have suggested an *a priori* focus on this subset of risk factors when this was not the case. Although not all factors remain important in our final model, nonetheless they were evaluated at suitable stages of the analysis for their importance as a risk factor for HRJL within the domain of risk factors in which they were considered (i.e. socio-demographic, lifestyle or employment related). If the Editor would like us to shorten tables 1 and 2 to only present data on the factors that feature in our final models then of course we are willing to effect these changes. However, we have retained our comprehensive tables for now which is an approach that we hope is particularly compatible with the online format of PLOS ONE.

Lastly, the HEAF study does include data on baseline morbidities from linkage with CPRD (Clinical Practice Research Database) records. This is a rich data source which will form the basis of future investigations in HEAF. The aim of our current paper was to really well describe with a clear gender focus a contemporary cohort of older workers, and to delineate the important sociodemographic, lifestyle and employment characteristics that are risk factors for health-related job loss, irrespective of consideration of specific morbidities which would of course be likely to increase the chance of exiting employment. With these factors now established, it is our intention to next explore the interplay between specific morbidities and the risk factors identified herein. For example, will the impact of these risk factors be amplified among people with eg mental health diagnoses or chronic musculoskeletal disorders, and the reverse question, will the impact of clinical diagnoses be amplified among people with the key risk factors identified in this paper? We outline this area for future work in the limitations section of our Discussion. We are mindful that our current paper is already substantial in content and length and therefore feel it is beyond the scope of the current paper to present the additional data on morbidities in HEAF.

We hope that our Introduction now provides a stronger justification of the gap in knowledge that our paper seeks to address and clarifies the novelty of our research. (See the 5^th^ and 6^th^ paragraphs of the Introduction for changes to the manuscript. Please note that additional references have been cited in the Introduction but our reference manager software did not enable all of these changes to be tracked in the text or the reference list.)

*5. The statistical methods should be available for review in the main body of the manuscript and not in an appendix.*
Thank you for this recommendation. We have replaced the Statistical Methods section in the body of the manuscript with the full version that was previously included as Appendix 2. We have re-numbered subsequent appendices, and references to them, accordingly.

*6. The measures of “obese” and “not obese” are less relevant as individuals age, when frailty is also a concern. This reviewer recommends re-categorizing into “underweight” “about the right weight” “overweight” and “obese” (or a minimum of 3 groupings) for improved analyses (Appendix 2)
7. If the data allow, this reviewer suggests making smoking status into three categories (never/previous/current) rather than two categories (never/ex or current) to improve analysis (Appendix 2).*
We thank the reviewer for these comments which we have addressed together. Interestingly, 10% of the HEAF men and women who classified themselves as ex-smokers had smoked up until only 3 years prior to the baseline questionnaire, and the median years smoked among ex-smokers was 22 for men and 19 for women. As such, many ex-smokers accumulated a smoking dose across a substantial period of time, and until fairly recently, such that in many cases the lifetime smoking dose of many ex-smokers was likely to rival that of many current smokers. As such, we felt it was not unreasonable to combine ex with current smokers to explore the impact of an overall lifestyle exposure to smoking. However, we fully accept that there is no absolute right or wrong way to classify variables such as smoking and BMI and we have therefore acted fully on the reviewer’s recommendations to change our analysis categorisations of these variables.

The changes that we have made throughout the manuscript are as follows: (1) S1_Appendix 1.pdf now specifies that the derived analysis groups for BMI and smoking status are ‘normal/underweight (<25kg/m^2^)’, ‘overweight (25-29.9kg/m^2^)’ and ‘obese/severely obese ≥30kg/m^2^’ and never/ex/current, respectively. We could not reliably examine ‘underweight (<18.5kg/m^2^)’ as a separate BMI category because only 6 men and 32 women who worked between HEAF baseline and two year follow-up were underweight. Please note that the changes to the S1_Appendix 1.pdf file are not tracked because if the paper is accepted for publication we were concerned that the S1_Appendix 1.pdf file might be used in that form; (2) We have removed the specification of the BMI categories from the full statistical methods (which has been included in the main text of the paper as detailed above) because we realised that this unnecessarily repeated information presented in Appendix 1; (3) Table 1 and its commentary have been updated to use the revised BMI and smoking variables; (4) Table 2 and its commentary have been updated to use the revised BMI and smoking variables. Use of the revised categorisation of never/ex/current smoking did not alter the result that in age-adjusted analyses, smoking was associated with HRJL among HEAF women (with a dose response relationship from never, to ex, to current smokers) but not men. Our conclusion that BMI is not a key player as a risk factor for HRJL among HEAF men was unaltered by analyses based on the revised BMI categorisation. However, age-adjusted analyses of the revised BMI categorisation in relation to HRJL did identify an increased risk of HRJL among women who were overweight or obese/severely obese in comparison with women of normal/underweight BMI; (5) Based on the results of the age-adjusted analyses for women, BMI was taken forward in our modelling strategy and merited inclusion as a lifestyle risk factor alongside smoking status in the final model for women (Table 3 and its commentary have been updated accordingly as has Fig 2). Whilst updating Table 3 we noticed that the number of HRJL events and person years at work by category of each exposure were based on univariate analyses. On reflection, so that the results presented in Table 3 are totally internally consistent, we have taken the opportunity to update these statistics so that they pertain to the samples of men and women who featured in the final models; (6) We have updated the fifth paragraph of our Discussion to detail BMI alongside smoking as a lifestyle risk factor for HRJL among HEAF women; (7) We have updated the Abstract statement about the lifestyle risk factors that were important for women.

*8. This reviewer is confused by the statement, “potential responder bias was examined by cross-tabulations and chi-squared tests of differences in the baseline characteristics”. Perhaps the authors intended to refer to potential recall error? (Appendix 2)*

Thank you for this comment and apologies for the confusion. We hope that we have now achieved clarity by amending the second paragraph of the revised statistical methods section to read as follows: ‘Differences in the baseline characteristics of HEAF respondents according to sex and whether or not they were in paid employment between baseline and 2-year follow-up were examined by cross-tabulations and chi-squared tests.’ We have also amended the first sentence of the Results section so that it now reads: ‘Table 1 describes the participants by sex and employment status between baseline and 2-year follow-up; 75% of men and 66% of women were employed at some point.’

*9. In the methods, you refer to “Cox proportional hazards models”, while the approach is identified as Cox’s proportional hazards models in the abstract. Either are correct but it’s important to be consistent throughout the manuscript (page 6, line 153).*

Thank you for noticing this inconsistency for which we apologise. We have edited the Abstract to read ‘Cox proportional hazards model’ in line with all other occurrences in the manuscript.

*10. What was the rationale for using this step-wise approach over a more parsimonious strategy? (page 6, line 151-161).*

Thank you for this question. The section under consideration is now located within lines 216-235 of the full statistical methods section in the tracked changes version of our revised manuscript. Apologies if our wording gave the impression that we used a step-wise approach, this was absolutely not the case. We did not choose a step-wise approach for statistical modelling because it hands the selection of the final model to a computer algorithm. Instead we advocate, and implemented, a researcher-driven approach to statistical analysis which was informed by the researcher’s knowledge about the likely interplay between the risk factors in question. We have added the following sentence to the fourth paragraph of our Statistical Methods and hope that this has clarified our approach: ‘We adopted a forward selection modelling strategy with the aim of identifying key risk factors for HRJL within each of the domains of socio-demographic, lifestyle, and employment characteristics before moving on to final mutually adjusted models.’

*11. How were missing data handled? and 22. Further discussion of the handling of missing data would be helpful here (page 20, lines 409-410).*

Apologies for not previously stating our approach to missing data, we have now specified in the full Statistical Methods section in the main body of the manuscript that we used a complete case analysis for our Cox proportional hazards models. We recognise that multiple imputation techniques can be considered for missing data, something that we now acknowledge in the limitations section of our Discussion with reference to Sterne JA, White IR, Carlin JB, Spratt M, Royston P, Kenward MG, et al. Multiple imputation for missing data in epidemiological and clinical research: potential and pitfalls. BMJ (Clinical research ed). 2009;338:b2393. A thorough application of multiple imputation methods to HEAF data is an area for future work.

*Results:
12. The sampling strategy should be included in the Methods section, rather than the Results section (page 6, lines 165-168).*

Thank you for this recommendation. We have moved the relevant paragraph from the start of the Results section to a section called ‘Study Sample’ at the end of the Methods section.

*13. This reviewer is confused how “mortgaging rather than owning their home outright” is evidence of a “healthy worker effect” (page 7, line 176).*

Thank you for this comment and apologies for our unhelpful choice of wording, the comparisons shown in Table 1 were not aimed at exploring healthy worker effects. We have amended the sentences in question so that they now read: ‘Table 1 shows that people who were employed at some point were on average more likely to be: younger; to have financial dependents; to mortgage rather than own their home outright; and to be doing some weekly physical activity.’

*14. This reviewer recommends omitting or greatly revising Figure 1, which contains more information than readers are likely able to process.*

We thank the reviewer for this comment but we would like to retain Fig 1 in its current form if the Editor will permit us to do so. The full statistical methods section is now included in the main body of the paper and it explains the classification of the workers according to their exit(s) from employment (or not) during the 2 years of follow-up. With this classification clearly set out, the reader is familiar with the groupings that we use in Fig 1. The graphs in themselves are simply then clustered bar plots for men and women separately, and we feel they provide an absolutely essential overview of not only the types of occupational groups of the HEAF participants by pattern of work exits, but also very importantly show the gender differences in the work done by HEAF men and women. We feel that our commentary in the Results section would be much diminished if we omitted Fig 1. Moreover, given that PLOS ONE is an online publication, a reader of our article would have the coloured figure fully available to them on screen and could display it in full page format if required. If the Editor would prefer us to include Fig 1 as a supplementary file rather than a core figure then we would of course be happy to make that change, but in some form we would really like to retain Fig 1 in our manuscript. We feel that it is this sort of detail that is so often omitted from debates about longer working lives, most particularly in acknowledging that women make up a substantial part of the older workforce now but are often working in very different types of jobs.

*Discussion/Limitations:
15. It is important to note that – due to the 2-year follow up only – many of these exits are likely not permanent and that a trajectory analysis would be helpful in future studies to capture these exits and potential re-entries.*

Thank you for this comment. It is a strength of HEAF’s longitudinal cohort study design that we are following participants up annually for at least five follow-ups, more if funding permits. As such we will be able to conduct the trajectory analyses of work exits and re-entries that the reviewer suggests and they will be fascinating.

For now, as reported in the Employment Exits sub-section of our Results section, we note that 324 (53.7%) of the men and 383 (55.7%) of the women who exited employment in the two year window did so with no return to work in this time frame, although some may of course eventually return to employment. In response to the reviewer’s comment we have added the following statement to the limitations paragraph of our Discussion and renumbered subsequent limitations that are discussed: ‘Third, we did not have a sufficient number of HRJL events to disaggregate analyses by permanent or temporary exits from employment, and moreover we could not be certain that exits that were not followed by a return to employment within only 2 years of follow-up would necessarily remain permanent. The ongoing annual longitudinal follow-ups of the HEAF cohort will generate valuable data on these older workers as they move through the retirement transition and we will be able to identify those who leave work permanently as opposed to those in whom there is a trajectory of work exits and re-entries.’

*15 (continued). It would be helpful to compare the prevalence of job exit among this sample with job exit in younger samples (page 17, lines 300-311).*

Thank you for this comment. To aid comparability of our results with those of other studies (published to date or in the future) that conduct a person-years employed analysis of their data we have expanded the start of the *Longitudinal analysis of risk factors for health-related job loss* section to read as follows: ‘The 2-year survival analysis file included 2418 men and 2614 women of whom 108 and 176 experienced a HRJL respectively. Rates of HRJL per 1,000 person-years employed were 25.0 (95%CI 20.7, 30.2) for men and 38.3 (95%CI 33.0, 44.4) for women.’

We are not aware of any publications which provide directly comparable and contemporary UK data with which we can compare the HEAF rates of HRJL. However, a search in response to the reviewer’s comment did locate the following paper which studied a limited range of predictors of HRJL via a postal survey of men aged 24-70 years in three rural areas of England and Wales: Solomon C, Poole J, Palmer KT, Coggon D. Health-related job loss: findings from a community-based survey. Occupational and environmental medicine. 2007;64(3):144-9. Solomon et al reported that ‘HRJL was reported by 1408 (13%) of the 10 559 men who had held long-term jobs. The incidence rose steeply with age for cardiorespiratory and neurological disorders, but for accidents and poisoning the trend was, if anything, in the reverse direction. An increase in incidence over time was most marked for musculoskeletal disorders and mental illness, and much less prominent for cardiorespiratory and neurological disease.’ Table 2 of Solomon’s paper reported incidence of first HRJL of a long-term job by 5-year age bands and calendar periods: for the calendar period 2000-2004, the incidences per 1,000 years were 13.3 for men aged 50-54, 17.0 for men aged 55-59, and 23.9 for men aged 60-64 (the incidences for men aged between 16 and 49 ranged between 3.7 and 9.2). There are many differences between HEAF and Solomon’s study: they are not contemporaneous and Solomon noted that rates of HRJL were increasing over time; HEAF includes urban and rural areas; HEAF collects data prospectively, Solomon’s postal survey was retrospective and relied on responders’ recall of occupational histories, activities and job exits; Solomon et al focussed their analysis on men in work at least a year which it could be argued might represent a subgroup of men in more stable longer term employment among whom premature exits from employment might have been lower than expected if the whole workforce is included, as is our approach in HEAF. Notwithstanding these differences, our HEAF rate of HRJL for men of 25.0 (95%CI 20.7, 30.2) per 1,000 years employed is entirely comparable with the incidence rate of 23.9 reported by Solomon et al for their oldest age group (60-64 years) studied in their most recent time period (2000-2004). Given the limitations of the comparison between Solomon’s study and HEAF we have not added it in to the manuscript but could do so if this would be helpful.

*16. This reviewer appreciated this discussion of policy implications on gendered findings. It is also important to note that many consider disability to relate to a “mismatch between a person and their environments”, which could include working environments. How could working environments be improved to maintain older workers, and to allow them to thrive? (page 17, lines 322 – page 18, lines 330).*

We are glad that the reviewer appreciated our discussion of policy implications on gendered findings. We are also grateful for the suggestion to expand our discussion around this area. Rather than postulating on specific physical changes that could be made to specific working environments, we have highlighted the importance of the role of the employer in enabling older workers to match their work with their life. Avoidance of undue mismatch between a person and their work environment, in its very widest sense, is indeed essential if older workers are to continue in employment. This paragraph of our Discussion now reads as below (please note that references are included in the tracked and clean versions of the main manuscript):

‘It is no surprise that work differs in men and women and yet macro level legislation such as changing the age of eligibility for a state pension takes no account of gender differences, or indeed any other heterogeneity in older workers. Our data pertain to a contemporary cohort of older workers in England who are progressing through their retirement transition at a time when recent legislation to raise and harmonise state pension age is taking effect: UK women born in the 1950s (HEAF participants were born between 1949 and 1963) have seen their age of eligibility for state pension rise from 60 to 66 years over one decade and further increases in state pension age are scheduled. HEAF women who reported HRJL during follow-up were particularly employed in educational, health and social care occupations. Employers should play a fundamental role in encouraging fuller working lives by enabling older workers to match their work with their life. Employer led initiatives to retain older workers include: encouraging and enabling flexible working which might include part-time or remote working, or variable start and finish times, which complement the caring responsibilities, physical capabilities and long-term health conditions that comprise the life context of each individual employee; providing opportunities for reskilling and redeployment to less physically demanding roles if desired by the employee; and listening to, engaging with, and responding to the needs of older workers so they feel a valued part of the workforce with opportunities and benefits equal to those of their younger colleagues. The relative importance of these employer based strategies for retaining older workers is likely to differ for employers with a mixed, predominantly male, or predominantly female workforce. Given that older working women are more likely to also have caring responsibilities than their male counterparts, our study suggests that flexible working policies might be of particular importance for the prevention of HRJL in sectors with predominantly female workforces such as education, health and social care.’

*17. The fact that the socioeconomic variables were no longer significant in the adjusted models does not necessarily suggest that proximity to retirement, physical activity, job satisfaction, and difficulty coping with the demands at work operated as mediators (page 18, lines 335-339). A mediation analysis is needed to make this claim and the model could very well be under-powered.*

Thank you for this comment and apologies that our statement was too strong, we certainly did not mean to imply that we had conducted a formal mediation analysis. We have altered the wording of the fourth sentence of the fourth paragraph of the Discussion section such that it now reads: ‘However, no socio-economic marker remained important in our fully adjusted model for men, suggesting that proximity to retirement, physical activity, job satisfaction and difficulty coping with the physical demands of work may mediate the impact of socio-economic position on HRJL among HEAF men.’

*18. The discussion preceding the statement, “Another way in which social inequalities might affect risk of HRJL” (page 19, line 734) concerns differential health behaviors by socioeconomic position. This reviewer recommends either (a) discussing more about the fundamental causes that could underlie those differential health behaviors, linking to inequalities or (b) changing “inequalities” to “factors”.*

Thank you for this comment, as recommended we have changed ‘inequalities’ to ‘factors’.

*19. In what direction do the authors anticipate the healthy participation effect might have influenced the results? (page 20, lines 370-372).*

Thank you for this comment which we believe refers to page 19, lines 370-372 of the original submission. We hope that we have increased clarity by rewording the sentences in question as follows: ‘A healthy participant effect, common to most cohort studies, may have also influenced our results if it diminished the range of lifestyle exposures arising among the study participants and limited our ability to discriminate them as risk factors for HRJL; however, many lifestyle risk factors were broadly similar among those who did any work between baseline and follow-up and those who did none, lessening this concern.’

*20. Please include a citation to the study (page 20, lines 394-397).*

Thank you, we have referenced the study earlier in this section.

*21. In what direction might the estimated incidence of HRJL be affected by the healthy participants bias? (page 20, lines 408-409).*

Thank you and apologies that our statement was not explicit. The sentence now reads: ‘Fourth, we observed a bias towards healthier participants of higher socio-economic position in the sample of workers versus non-workers during the two year follow-up; a bias arising in most cohort studies. Although the estimated incidence of HRJL might be lowered by this bias, the principal relationships that we have explored between participant characteristics and HRJL were internal to the sample.’

***Reviewer #2:*** *Revision Article

-The article PONE-D-20-01352 aimed to explore risk factors for health-related job loss over 2 years of follow-up, among a cohort of contemporary older workers in England. To this purpose the authors analyzed labor-force transitions of 5,032 individuals aged 50-64 (at baseline), using two rounds of the Health and Employment after Fifty (HEAF) panel study, and employing semi-parametric survival analysis.
-The study topic is appealing, timely and relevant, given the focus on a relevant life transition in ageing societies. However, after reading the manuscript twice I have multiple major concerns regarding the introduction of the study, the discussion of previous literature, and the novelty of this article, which if they are not seriously considered, I would suggest to the Editor of PLOS ONE not to publish this manuscript.*

We thank the reviewer for their comments and we are delighted that they agree that our study topic is appealing, timely and relevant. We apologise for the shortcomings that the reviewer identified and we value the opportunity to revise our manuscript. We feel that the extensive changes that we have now made to our manuscript (detailed in point by point responses to the reviewers) have greatly improved it and we thank the reviewer for their part in this process.

*Introduction
-The most notorious weakness of the introduction is that it does not identify appropriately a knowledge gap in this study field that needs to be addressed. Author actually mention that a “focus on identification of the wider drivers of health-related job loss, irrespective of specific illnesses or diagnoses, therefore offers the potential to inform the development and implementation of workplace strategies to encourage and enable work to older ages.” However, this is not really original in the field of retirement and health studies. So, from my point of view, it is not clear why the main study subject (i.e., risk factors for health-related job loss) deserves to be explored, challenged, or revisited. I am not saying this is not an important subject, but rather that its importance is not clearly discussed or explained to the reader.
-Also, the discussion held in the introduction is not really coherent. Specifically, authors provide first a discussion on extended working policies to then mention that health is a major determinant for early labor market exits. Given that the allowed number of words is limited, if the main topic of this research is the determinants of health-related job loss (which leads individuals to early exit from the labor market), I would expect a more focused theoretical discussion on early retirement due to health reasons, and particularly on what remains unexplored in this field.*

We thank the reviewer for these comments and apologise for the brevity of our Introduction. We were mindful that our manuscript is relatively long because we provide a comprehensive exposition of our research, but we now realise that in our bid for brevity we omitted some of the detail from the Introduction which justifies the novelty of our work and places it in the wider context of the academic literature.

Our principal focus is indeed on health-related job loss and our literature review focussed on this outcome. We have amended our Introduction to make this clear and have now clearly made the case that there are no previous studies which have explored a comprehensive set of risk factors for health-related job loss in a UK setting. Every country is unique in terms of its political and social contexts, as such, although some findings may be generalizable between different countries, we feel that our UK based study is extremely important and has the ability to greatly inform the ongoing debate on policies designed to extend working lives.

We hope that our Introduction now provides a stronger justification of the gap in knowledge that our paper seeks to address and clarifies the novelty of our research. (See the 4^th^ and 5^th^ paragraphs of our revised Introduction for changes to the manuscript. Please note that additional references have been cited in the Introduction but our reference manager software did not enable all of these changes to be tracked in the text or the reference list.)

*Data & Methods
-There is no justification provided about why women and men are separated in the analysis.*
We thank the reviewer for pointing out that we had not explicitly justified why we analyse men and women separately. We have amended the first paragraph of the Statistical Methods so that it now clearly links between the literature that we reference in the Introduction and our chosen analysis strategy, and now reads: “Men and women were analysed separately throughout; this analysis strategy was decided *a priori* because work and its social context typically differ between men and women, and previous research has called for investigation of gender differences in risk factors for health-related job loss. Analyses were conducted using the Stata statistical software package (release 15).”

*-There is no test of the main assumption of the statistical models used in this research: proportional hazard of predictors over the dependent variable.*
Thank you for this comment. We have now amended the final section of the Statistical Methods section so that it reads: “Tests of the proportional hazards assumption in the final models were based on Schoenfeld residuals and implemented using the *estat phtest* command in Stata. Log-log plots were also used to graphically assess the proportional-hazards assumption.”

*Discussion
-I believe authors once again did not express clearly what the novelty or originality of this study is. Authors for instance mention: “Our work is novel in considering self-reported exit from employment mainly or partly for health reasons”; but this is only partly true.*

Apologies if we over stated the novelty of our work, this was not intended. Our expanded Introduction hopefully now makes it clearer that our study addressed a gap in knowledge by considering a wide panel of risk factors for self-reported health-related job loss in a contemporary cohort of older workers in England. In response to this specific comment, we have also amended the first few lines of our Discussion, and much of the second paragraph, to emphasise the value of our approach of considering self-reported health-related job loss but we have been careful not to state that our work is novel, rather we emphasise its value.

*Then they state:“Our data are novel in pertaining to a contemporary cohort of older workers who are progressing through the retirement transition at a time when recent legislation to raise and harmonise state pension age is taking effect”;*

Thank you for this comment. We have removed the statement that our data are novel and edited the first sentence highlighted by the reviewer to read: “Our data pertain to a contemporary cohort of older workers in England who are progressing through their retirement….”

*however, I am highly doubtful on whether the recent modifications in the retirement legislation affected the cohorts studied in this research.*

In the UK the default retirement age of 65 years was abolished in 2011 and the coalition government’s 2011 Pensions Act brought the new qualifying age of 65 for women forward to 2018 (it had originally been planned to be phased in between 2010 and 2020). The qualifying pension age for both men and women will be raised to 66 by October 2020. The Pensions Act 2014 brought the increase in the State Pension age from 66 to 67 forward by eight years. The State Pension age for men and women will now increase to 67 between 2026 and 2028. Under the Pensions Act 2007 the State Pension age for men and women will increase from 67 to 68 between 2044 and 2046 although this could change as a result of review (the Pensions Act 2014 provides for a regular review of the State Pension age, at least once every five years. The review will be based around the idea that people should be able to spend a certain proportion of their adult life drawing a State Pension.) Our HEAF participants were aged 50-64 at baseline in 2013-2014 so have birth years ranging between 1949 and 1963. As such, we do feel that the pensions landscape has changed markedly for our HEAF participants as they have moved into the later phase of their working life and we do feel that the recent modifications in the retirement legislation have, and are continuing, to affect our HEAF participants. We have edited the sub-clause in the sentence highlighted by the reviewer to make this clear: “UK women born in the 1950s (HEAF participants were born between 1949 and 1963) have seen their age of eligibility for state pension rise from 60 to 66 years over one decade and further increases in state pension age are scheduled.” In the interests of brevity we have not included any of the legislative background information detailed above but are happy to do so if the Editor would like us to do so.

*-Finally, from my perspective, the discussion about policy contributions of this research does not provide any original idea to the debate on extended working lives. Previous retirement research has provided very similar reflections during the last 10-12 years.*

Thank you. These comments encouraged us to reflect more clearly on what it is that we feel are the important messages for employers and policy-makers. We have included a whole new paragraph as follows in the Discussion (please note that references are included in the tracked and clean versions of the main manuscript):

‘Whilst our findings begin to hint at the complexity of enabling work to older ages, there are some key messages for employers and policy-makers. It seems that employers would be well-advised to take a nuanced approach to retaining older workers, considering the needs of male and female workers separately and evaluating their perception of their physical and mental capability in relation to their assessment of the job demands. Attempts to measure, and improve, rates of job satisfaction could also lead to increased retention of older workers. Achieving this might, for example, involve consultation with older workers’ representatives and implementing suitable and inclusive policies around flexible working to enable caring responsibilities. For policy-makers, the message is clear that blanket policies of raising the age of eligibility for state pension and prohibiting age discrimination will not be universally effective at extending working lives: it is generally easier to remain in sedentary jobs than it is to stay working in those which are physically very demanding. Moreover, our results hint that the burden of HRJL among older workers will not be equal, and that those who are socioeconomically disadvantaged and struggling financially (for example as a consequence of the accumulated effects of lower pay, not owning their home, and a lack of private pension arrangements) will be more likely to need other governmental financial support through welfare benefits before they are old enough to claim their state pension. Going further however, what may be needed by policy-makers is a cultural shift from regarding capability to remain in work to older ages as a challenge for occupational health departments and employers to fix, to a recognition that a long working life is something that needs to be addressed and planned for by individuals, employers and public health policies throughout a person’s life. In order to work later in life, mental and physical capacity needs to be preserved and these are both influenced by factors that operate throughout the lifecourse. Our results that risk of HRJL is related to physical activity among men, and smoking and BMI among women, show that lifecourse health behaviours influence work ability beyond the age of 50 years. Employers and policy-makers could usefully consider ways in which to promote healthy behaviours throughout the lifecourse both at work and outside of the workplace, thereby benefitting all.’

*More importantly, in this section I expected an in-depth reflection on how the study findings might help scholars as well as also policy makers to approach differently to the understanding of the closed relationship between work, health, retirement and sociodemographic factors (such as genders, educational levels, or cohorts). But this is unfortunately not achieved.* 

Thank you. In response to this comment, in addition to the inclusion of the substantial section detailed above, we have revised the final paragraph of our paper to read as follows:

# ‘In summary, our results emphasise that the employment contexts and characteristics of contemporary older male and female workers are different and that characteristics such as job satisfaction and perceived ability to cope with the physical and mental demands of work are key determinants of HRJL. The implications are twofold; first for future research, and second for workplace interventions and policies aimed at extending working life. We recommend that future studies of older workers collect data longitudinally with time-varying covariates and consider sex differences, and investigate the predictive, confounding, or mediating role of a wide panel of potential risk factors for HRJL, with psychosocial work characteristics key amongst these. A next step will be to explore the interplay between the important factors identified in this study with specific morbidities also known to have a marked impact on premature exit from the workforce such as musculoskeletal disorders and mental health conditions. Our results also support calls for the development of policies and workplace interventions which: take a flexible, person-centred approach to extended working life, recognise the importance of psychosocial work characteristics, and acknowledge heterogeneity between employees, occupations, and employment settings.’
